# Challenges with Pain Treatment for Rural Older Adults: Family Caregivers' Views

**DOI:** 10.1093/geroni/igab046.3220

**Published:** 2021-12-17

**Authors:** Hyunjin Noh, Cho Rong Won, Zainab Suntai

**Affiliations:** 1 University of Alabama, Tuscaloosa, Alabama, United States; 2 University of Alabma, Tuscaloosa, Alabama, United States

## Abstract

Family caregivers face various challenges in assisting older adults experiencing pain and difficult symptoms. Living in rural areas poses additional obstacles to their caregiving. The purpose of this study was to explore family caregivers’ lived experiences in caring for older adults with pain and discomfort in rural communities. A qualitative research design was adopted to capture the common essence of participants’ experiences through a phenomenological method. Purposeful sampling was used, and the participant criteria was: age 18+, have good thinking skills, resident of Alabama, provide unpaid assistance to a family/relative who has chronic/serious health conditions and experienced pain/discomfort in the last 3 months. Ten participants were recruited from rural counties of Alabama. Individual semi-structured interviews were conducted via phone and were recorded and transcribed verbatim. Inductive, thematic analysis of the data revealed themes in five categories: 1) impact of pain (physical and psychological/emotional toll), 2) coping strategies (faith/contentment with life/logistical adaptation), 3) impact of Covid-19 (physical health/social interaction/mental health/added caregiving), 4) challenges in pain treatment (transportation (time/distance/driver/cost) and non-transportation related problems (healthcare provider issues/health insurance/financial burden)), and 5) suggestions (transportation-related (more transportation options/tailored services) and non-transportation-related support (home-based services/better health insurance coverage)). Findings of this study highlight rural family caregivers’ unique experiences in assisting older adults’ access to pain treatment, particularly during the Covid-19 pandemic. Policy- and program-level intervention is called for to increase individualized transportation options, improve health insurance coverage, and expand financial support for rural older adults experiencing pain and their caregivers.

